# Coixendide efficacy in combination with temozolomide in glioblastoma and transcriptome analysis of the mechanism

**DOI:** 10.1038/s41598-023-41421-w

**Published:** 2023-09-19

**Authors:** Zhenran Zhao, Lei Zhang, Xiaohan Zhang, Yong Yue, Shengchen Liu, Yanan Li, Xiang Ban, Cuizhu Zhao, Peng Jin

**Affiliations:** 1 Neurosurgery, The Affiliated Hospital of Qingdao University, Qingdao, 266000 Shandong China; 2 Neurosurgery, Linyi Traditional Chinese Medical Hospital, Linyi, 276000 Shandong China; 3 College of Agronomy, Northwest A&F University, Yangling, Xianyang, 712100 Shaanxi China

**Keywords:** Oncology, Cell signalling

## Abstract

The purpose of this study was to explore the role of coixendide (Coix) combine with temozolomide (TMZ) in the treatment of Glioblastoma (GBM) and explore its possible mechanism. CCK-8 was used to determine the inhibitory rate of *Coix group*, *TMZ group* and *drug combination group* on GBM cells, and the combination index (CI) was calculated to determine whether they had synergistic effect. Then RNA was extracted from each group, transcriptome sequencing was performed, and differentially expressed genes (DEGs) were identified. The possible mechanism was analyzed by GO enrichment analysis and KEGG enrichment analysis. The CI of Coix and TMZ indicating a synergistic effect when TMZ concentration is 0.1 mg/ml and Coix concentration is 2 mg/ml. Transcriptome sequencing analysis showed that interferon (IFN) related genes were down-regulated by Coix and up-regulated by TMZ and combined drugs, however, the up-regulation induced by combined drugs was less than that of TMZ. Besides IFN related genes, cholesterol metabolism pathway were also been regulated. Coix and TMZ have synergistic effects in the treatment of GBM at certain doses. RNA-Seq results suggested that the abnormal on genetic materials caused by DNA damage induced by TMZ treatment can be sensed by IFN related genes and activates antiviral IFN signaling, causing the activation of repairing mechanism and drug resistance. Coix inhibits IFN related genes, thereby inhibits drug resistance of TMZ. In addition, the activation of ferroptosis and the regulation of DEGs in cholesterol metabolism pathway were also contributed to the synergistic effects of Coix and TMZ.

## Introduction

Glioblastoma (GBM) is the most malignant tumor in astrocytoma, and the WHO grade reaches grade IV^1^. The centerpiece of GBM treatment is surgery, followed by radiation and adjuvant chemo-therapy^2^. At present, the main drug treatment for GBM is temozolomide (TMZ), but GBM is more likely to develop resistance to TMZ than other types of gliomas, and the median survival time of patients after treatment has not been significantly prolonged^3^. The activation of DNA repair pathway is the main mechanism of this phenomenon, which repairs DNA damage induced by TMZ and prevents cells from entering apoptosis. Several new drug resistance mechanisms also have been found, such as miRNA participation, drug efflux transporter, gap junction activity, the emergence of glioma stem cells and the up-regulation of cell survival autophagy.

Researchers are constantly looking for drugs that can reverse drug resistance of TMZ, and many have been found. Some of them play a role in regulating the expression of O^6^-methylguanine-DNA methyltransferase (MGMT), such as Cordycepin, Levetiracetam (LEV), NBM-BMX(BMX), S-nitroso-*N*-acetylpenicillamine (SNAP); Other drugs have effect on the process of apoptosis, these include Chloroquine, Lovastatin, Romidepsin (FK228), Delta(9)-Tetrahydrocannabinol (THC), Tamoxifen (TMX), Hypericin; There are also some drugs that act in other aspects, such as Metformin (MET) and BIX01294 inhibiting glioma stem cells (GSCs), Isofuranodiene (IFD) and Difluoromethylornithine (DFMO) inducing cell cycle arrest, Afatinib inhibiting EGFRvIII/AKT, EGFRvIII/JAK2/STAT3, and focal adhesion kinase (FAK) signaling pathways, and Psammaplin C overcoming P-glycoprotein-mediated TMZ resistance in GBM^4^. Some plant extracts, such as European celery leaf extract, also have been reported to have synergistic effect on TMZ^5^.

Coixendide (Coix) is an active extract of the Coixlacryma-jobi seed (family: Cramineae). It is the main active ingredients of Kanglaite (KLT), a biphasic broad-spectrum anti-cancer drug, and an oily components extracted from Coixlacryma-jobi^6^. KLT is one of the ten Chinese herbal injections used in cancer treatment that covered in the Chinese national essential health insurance program. It shows synergistic effects in combination with radiotherapy and chemotherapy in the treatment of a variety of tumors^7^.However, whether it has curative effect on GBM and whether it can be combined with TMZ to improve the therapeutic effect of TMZ has not been studied.

## Materials and methods

### Cell lines and culture conditions

The Human GBM cell line U251 MG was purchased from National Collection of Authenticated Cell Cultures of China. Cells were cultured with Dulbecco’s Modified Eagle Medium in an incubator with a humidified atmosphere at 37 °C containing 5% CO2.

CCK-8 kit (Biological Industries, BI, Israel), dimethyl sulfoxide (DMSO, BI, Israel), Dulbecco’s Phosphate-Buffered Saline (BI, Israel), DMEM medium (BI, Israel), fetal bovine serum (BI, Israel), trypsin enzyme (BI, Israel), trypan blue (BI, Israel).

### CCK-8 drug inhibition rate experiment

KLT injection (Zhejiang Kanglaite Pharmaceutical Co., Ltd, Zhejiang, China) was used for Coix treatment. KLT injection were diluted with culture medium to the concentration of 30 mg/ml, 20 mg/ml, 10 mg/ml, 7.5 mg/ml, 5 mg/ml, 4 mg/ml, 3 mg/ml, 2 mg/ml, 1 mg/ml and 0.5 mg/ml. TMZ (Jiangsu Hengrui Pharmaceuticals Co., Ltd. Al. Jiangsu, China) were dissolved in sterilized water at the concentration of 2.5 mg/ml, and diluted with culture medium.

The cell concentration of U251 human glioma cell line was seeded at a density of 5000 cells/well in 96-well plates and incubated for 24 h. Then, the cells were incubated with culture medium containing different concentrations of TMZ and Coix for 24 h. CCK-8 was added into the culture medium to prepare the solution of CCK-8 (the culture medium was 1:10) and Added 100 μl solution per well, i.e. 10 μl CCK-8 per well. The 96 well plate was wrapped with tin paper and incubated in the incubator for 2 h. The absorbance at 450 nm was measured by enzyme-labeled instrument.

### Sample preparation for RNA sequencing

The cell plating and dosing steps are the same as those of CCK-8 experiment. After 24 h of dosing and incubation, observe the cultured cells under the microscope to confirm the integrity of the cells. The adherent cells were cleaned twice with precooled D-PBS, an appropriate amount of Trizol was added, the cells were fully lysed by blowing with a pipette, and the lysate was transferred to 1.5 ml enzyme-free tube and stored at − 80 °C.

### Transcriptome analysis process

The raw data is filtered, and the filtered high-quality sequence (clean data) is aligned to the reference genome of the species. According to the comparison results, the expression of each gene was calculated. The differential expression of genes was analyzed by DEseq. The conditions for screening differentially expressed genes were as follows: the multiple of expression difference |log2foldchange|> 1, and the significance P-value < 0.05. TopGO is used for GO enrichment analysis.

### Quantitative RT–PCR analysis

The extracted RNA was used in the same batch as RNA-seq. First-strand cDNA was synthesized by using the PrimeScript TM RT reagent Kit with gDNA Eraser (Takara). qPCR was performed in three replicates using SYBR Premix Ex TaqTM II (Takara) on an ABI QuantStudio 7 Flex Real Time PCR system. β-actin were used as internal standard. The Ct value was calculated by the 2−∆∆CT method. All primers are listed in Supplementary Table “primer”.

Detailed experimental materials and methods can be found in the Supplementary Documents (Materials and method.docx).

## Results

### Synergistic effect of TMZ combined with Coix

**Figure 1:**
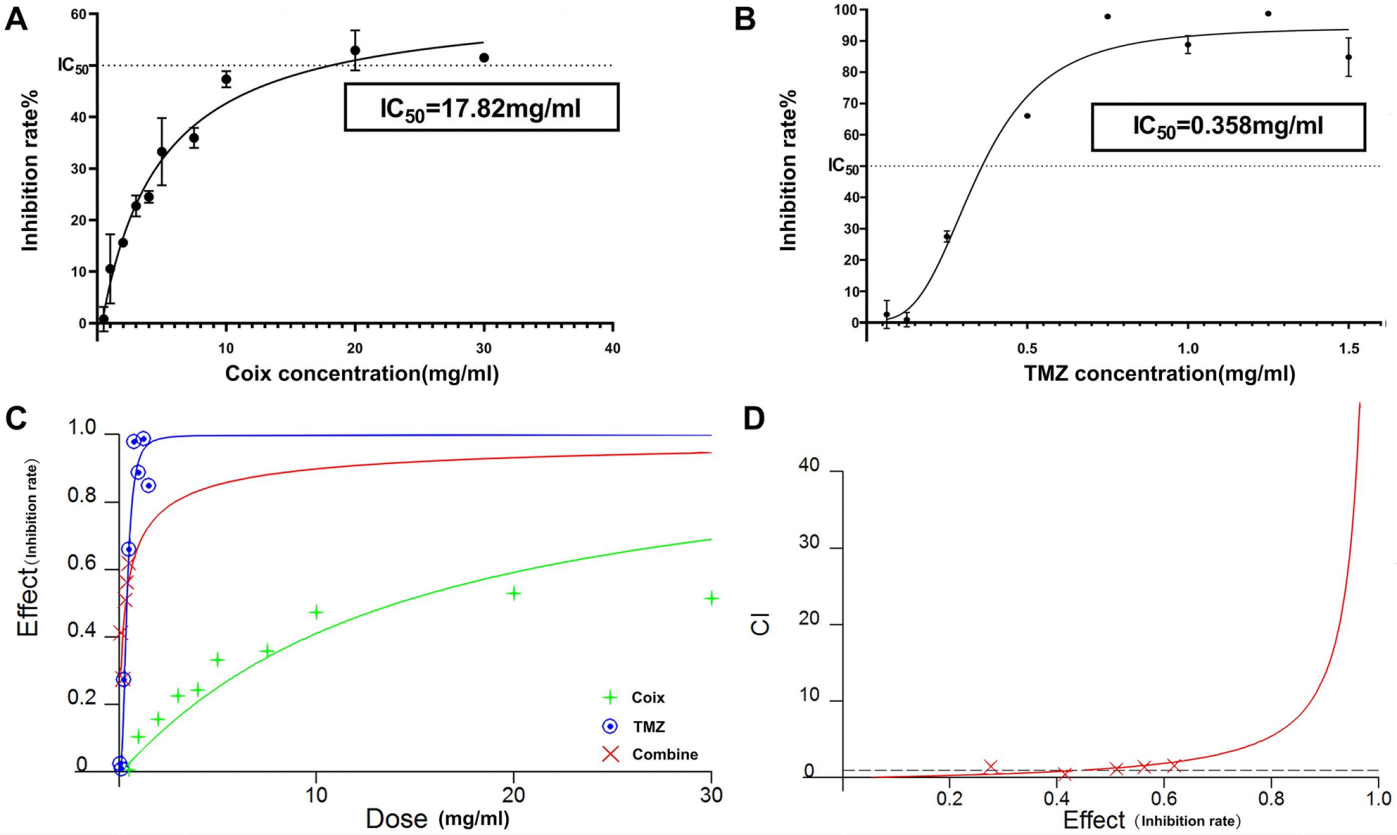
(**A**) Dose–effect curve of Coix group. The abscissa is the dose of Coix, and the ordinate is the growth inhibition rate of Glioblastoma cells. IC_50_ (= 50% inhibiting concentration): Drug concentration with a 50% inhibition rate. (**B**) Dose–effect curve of temozolomide (TMZ) group. The abscissa is the dose of TMZ, and the ordinate is the growth inhibition rate of Glioblastoma cells. (**C**) Dose–effect curve of TMZ group, Coix group, Combine group. The abscissa is the dose of each group, and the ordinate is the growth inhibition rate of Glioblastoma cells of each group. The blue line represents Dose–effect curve of TMZ group. The green line represents Dose–effect curve of Coix group. The red line represents Dose–effect curve of Combine group. (**D**) Fa-CI plot of Combine group. The abscissa is the Fa (Fa is the fraction affected by the dose), which represents inhibition rate of Combine group in this Figure. The ordinate is the combination index (CI) of Combine group. (When the CI is less than 1, the drug exhibits synergistic effects. When CI = 1, the drug exhibits additive effects. When CI is greater than 1, the drug exhibits antagonistic effects). The image (**A,B**) were generated by GraphPad Prism (v9.4.0), URL: https://www.graphpad.com, the image (**C,D**) were generated by calcusyn (v2), URL: https://wpkg.org/index.php?title=CalcuSyn&oldid=10764.

To find out whether Coix have synergistic effect on TMZ in the treatment of GBM, we tested the individual and combinatorial effects of Coix and TMZ on glioma cell U251 line by examining the numbers of living cells through Cell Counting Kit-8 (CCK-8) assays. First, dose effect curves of individual TMZ and Coix were drawn respectively according to the results of CCK-8 assays to reveal the individual effect of the two drugs and to determine the concentration ratio of the combined drug. The results showed that Coix and TMZ both inhibited the proliferation of U251, and the TMZ had a stronger inhibitory effect than Coix. According to the IC_50_ of Coix (17.82 mg/ml) and TMZ (0.358 mg/ml) (Fig. 1A,B), the concentration ratio of the combined drug was determined as Coix: TMZ = 1:20.

Then, the mixed drug was applied on U251 to measure the combinatorial inhibition rate of the two drugs. The results were input into the software calcusyn.2 to calculate combined index (CI) by using the median formula (Fig. 1C,D)^8^. The results showed that when the concentration of TMZ is 0.1 mg/ml and the concentration of Coix is 2 mg/ml, the CI of the two drugs is less than 1, indicating a synergistic effect (Fig. 1D). Under this concentration, the drug reduction index (DRI) of TMZ was 3.266, Therefore, TMZ combined with Coix have synergistic effect and could reduce the dosage of TMZ (DRI test was also carried out for Coix, and the inhibition rate was 0%, excluding the effect of solvent on the drug) (Table 1, Supplementary Table 1).

**Table 1:** CI of mixed drugs at different doses.

TMZ concentration (mg/ml)	Coix concentration (mg/ml)	Inhibition rate%	CI
0.1	2	41.4193	0.504
0.2	4	27.5726	1.490
0.3	6	51.0656	1.201
0.4	8	56.3093	1.420
0.5	10	61.8352	1.563

### Overview of RNA-seq experiments

**Figure 2:**
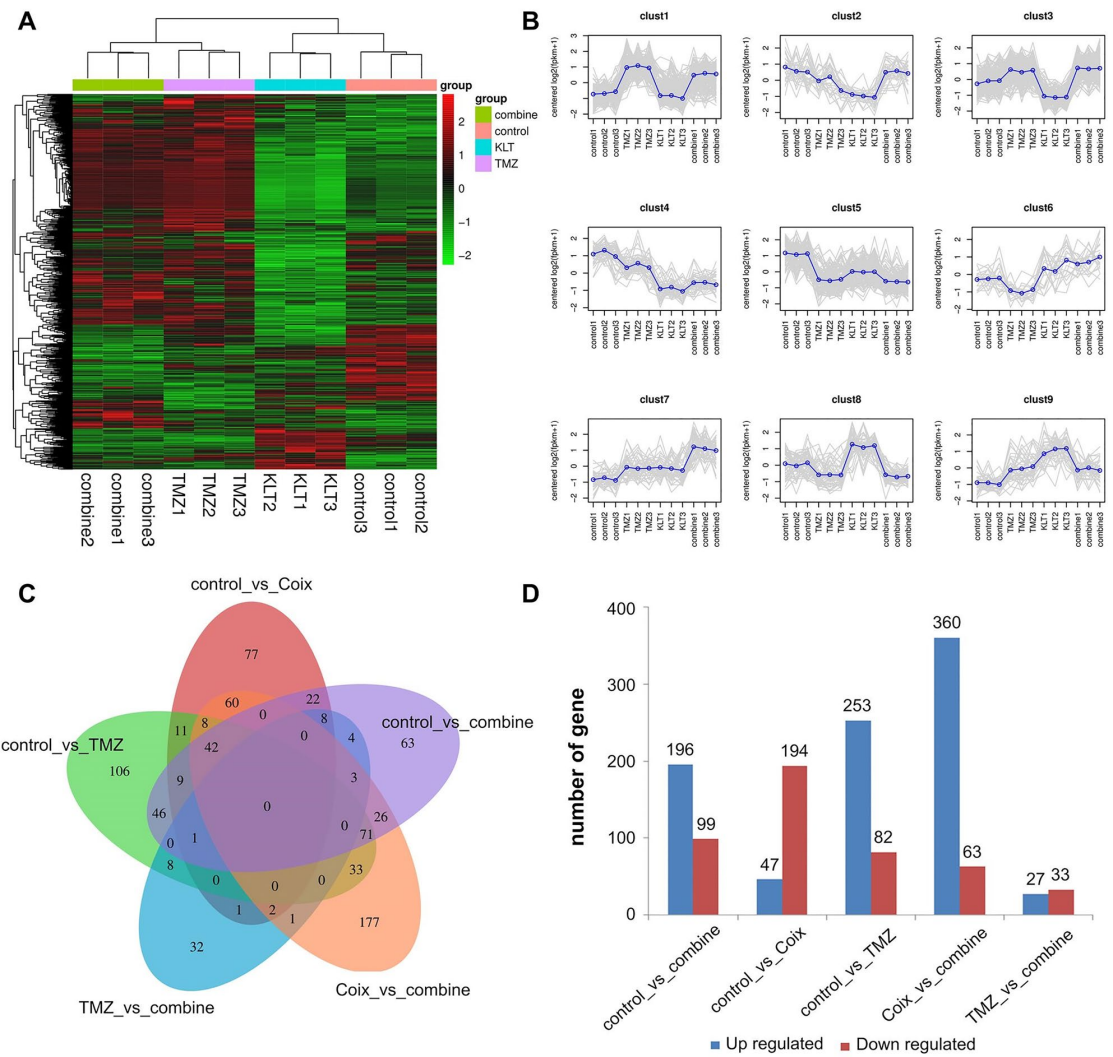
(**A**) Heatmap of all shared differentially expressed genes (DEGs) expression level in four treatment groups. Genes are represented horizontally, and each column is a sample. Red indicates high expression genes, and green indicates low expression genes. (**B**) Nine results of cluster analysis. The gray line shows the expression pattern of genes in each cluster, and the blue line represents the average expression amount of all genes in the cluster in the sample. (**C**) Venn diagram of DEGs among different groups. The sum of the numbers in each circle represents the total number of differential genes in the comparison combination, and the overlapping part of the circles represents the common differential genes between the two comparison groups. (**D**) Histogram of DEGs among different groups. The abscissa represents the comparison group for difference analysis, and the ordinate represents the number of difference genes. Red bar indicates the number of up-regulated genes and blue bar indicates the number of down regulated genes. The images were generated by: DESeq (v1.38.3), URL: https://bioconductor.org/packages/release/bioc/html/DESeq2.html.

To comprehensive the mechanism of the synergistic effect of TMZ combined with Coix against GBM proliferation, transcriptome analysis was carried out for U251 with or without Coix, TMZ, and Combination treatment. An average number of 44,103,525 clean reads were generated with 97% Q20, and 19,973 genes were assembled. Genes that were differentially expressed (DEGs) among samples were assessed using a false discovery rate (FDR)-corrected probability value of q < 0.05 and at least a twofold change. A total number of 16,391 transcript genes were identified in this study, among which 1354 DEGs were annotated in five categories and assigned to Go terms and pathways of the KEGG database (Figs. 2C,4B)^9–11^. Nine regulation patterns were recognized according to the gene expression differences among the four groups (Fig. 2A,B). Cluster1: 295genes. Cluster2: 27 genes. Cluster3: 173 genes. Cluster4: 46 genes. Cluster5: 166 genes. Cluster6: 18 genes. Cluster7: 40 genes. Cluster8: 51 genes. Cluster9: 36 genes. Cluster1 contains the most key genes. Detailed genetic Information can be found in the attached document (cluster Division and annotation.xlsx).

### Interferon related genes regulated by Coix treatment

**Figure 3:**
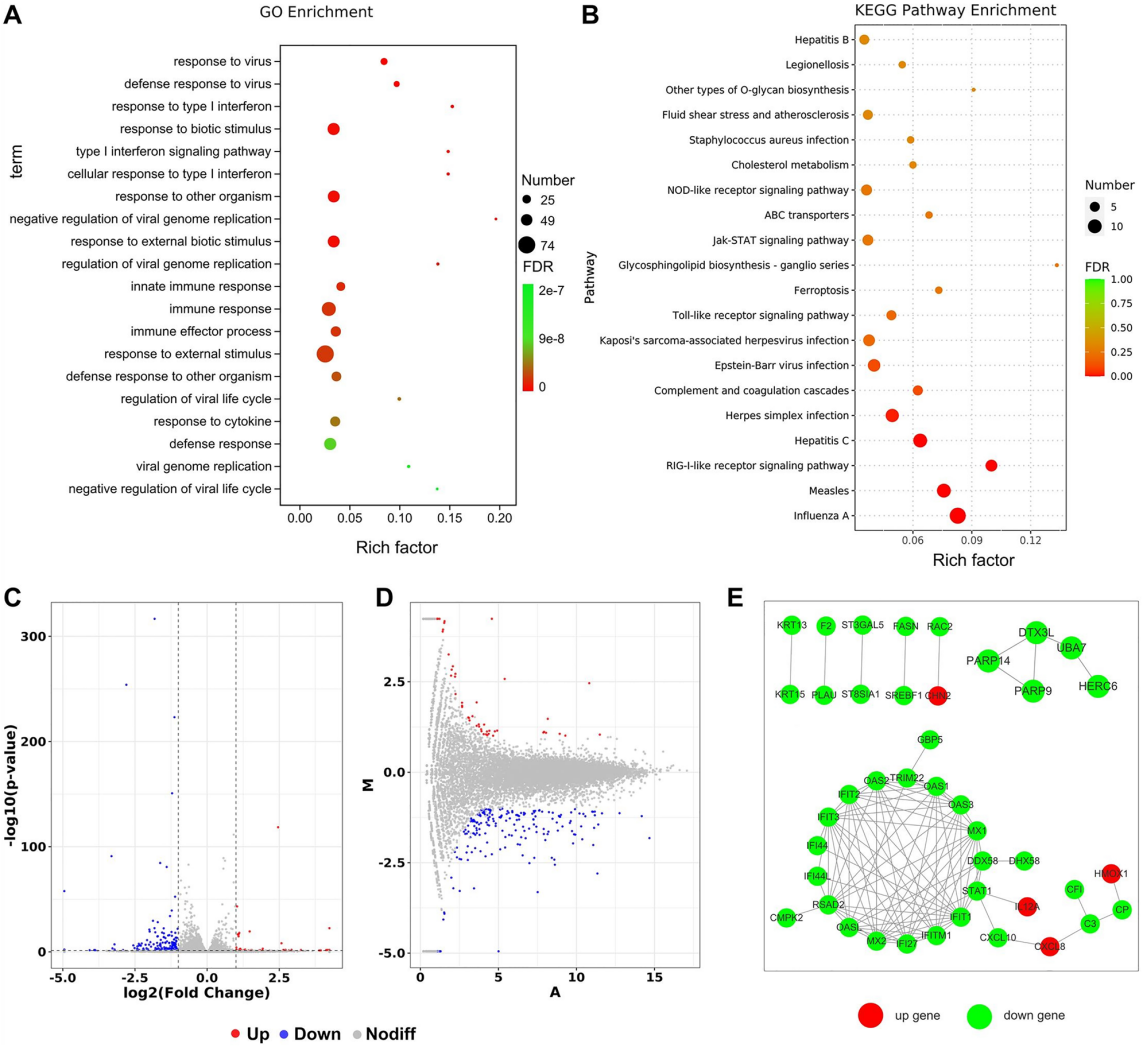
(**A**) GO enrichment of differentially expressed genes (DEGs) between Coix group and control group. The abscissa is rich factor, which refers to the ratio of the number of differential genes enriched in the GO term to the number of genes annotated. The larger the rich factor, the greater the degree of enrichment. The ordinate is the enriched term. The color of bubbles represents the value of false discovery rate (FDR). The general value range of FDR is 0–1. The closer to zero, the more significant the enrichment is. The size of bubbles indicates the number of enriched differential genes. (**B**) KEGG pathway enrichment of DEGs between Coix group and control group. The abscissa is rich factor, which refers to the ratio of the number of differential genes enriched in the pathway to the number of genes annotated. The larger the rich factor, the greater the degree of enrichment. The ordinate is the enriched pathway. The color of bubbles represents the value of FDR. The general value range of FDR is 0–1. The closer to zero, the more significant the enrichment is. The size of bubbles indicates the number of enriched differential genes. (**C**) Volcano plot showing DEGs between Coix group and Control group. The abscissa is log2FoldChange and the ordinate is − log10 (P-value). The two vertical dashes in the figure are 2 times the expression difference threshold. The horizontal dotted line is P-value = 0.05 threshold. The red dot indicates the up-regulated genes, the blue dot indicates the down regulated genes, and the gray dot indicates the non-significant differentially expressed genes. (**D**) M-versus-A plot (MA plot) showing DEGs between Coix group and Control group. The abscissa is log2 (A × B), (**A,B**) respectively represent the expression amount of genes in the two samples, and the ordinate is log2 (A/B). The red dot indicates the up-regulated genes, the blue dot indicates the down regulated genes, and the gray dot indicates the non-significant differentially expressed genes. (**E**) Network of DEGs involved between Coix group and control group. The dots are genes (corresponding proteins), where red represents up-regulated genes and green represents down regulated genes. The dots linked by gray lines are homologous proteins. The image (**A**) was generated by: topGO (v2.50.0), URL: https://www.bioconductor.org/packages/release/bioc/html/topGO.html. The image (**B**) was generated by: clusterProfiler (v4.6.0), URL: https://www.bioconductor.org/packages/release/bioc/html/clusterProfiler.html, images (**C,D**) were generated by: ggplots (v2), URL: https://www.r-project.org/, image (**E**) was cited from STRING database, URL: https://string-db.org.

Under 2 mg/ml Coix treatment, 241 DEGs were found between Coix group and control group, including 47 up-regulated and 194 down-regulated genes (Figs. 2D, 3C,D). These DEGs distributed in 193 pathways according to KEGG pathway enrichment analysis, and the top five enriched pathways are overrepresented (FDR less than 0.05). The five pathways include four viral infectious disease associated pathways (Influenza A, Measles, Hepatitis C, Herpes simplex infection) and one immune system pathway (RIG-I-like receptor signaling pathway). In fact, among the top 20 significantly enriched pathways, 13 of them are either infectious disease associated pathways or immune system pathways (Fig. 3A,B), indicating Coix treatment inhibits GBM through acting on body's defense system against infection. Network of DEGs involved between Coix group and control group is shown in Fig. 3E.

Further investigation found that most of the DEGs in the top five enriched pathways are overlapping and are associated with interferon (IFN), such as IL-12, IL-8, STAT1, MX2, OASs, IFIH1, CXCL10, RSAD2. This suggesting that the transcript change of IFN associated genes were the reason of infectious disease associated pathways and immune system pathways overrepresented in the Coix treatment, which indicating that IFN related defense biological processes were crucial to the inhibiting effect of Coix against GBM. This suggesting that the transcript change of IFN associated genes were the reason of infectious disease associated pathways and immune system pathways overrepresented in the Coix treatment, which indicating that IFN related defense biological processes were crucial to the inhibiting effect of Coix against GBM. This deduction is supported by the result of GO analysis which are all related to IFN and virus infection defense (Fig. 5A,B).

### Opposite regulation of interferon related genes between TMZ and Coix treatment

**Figure 4:**
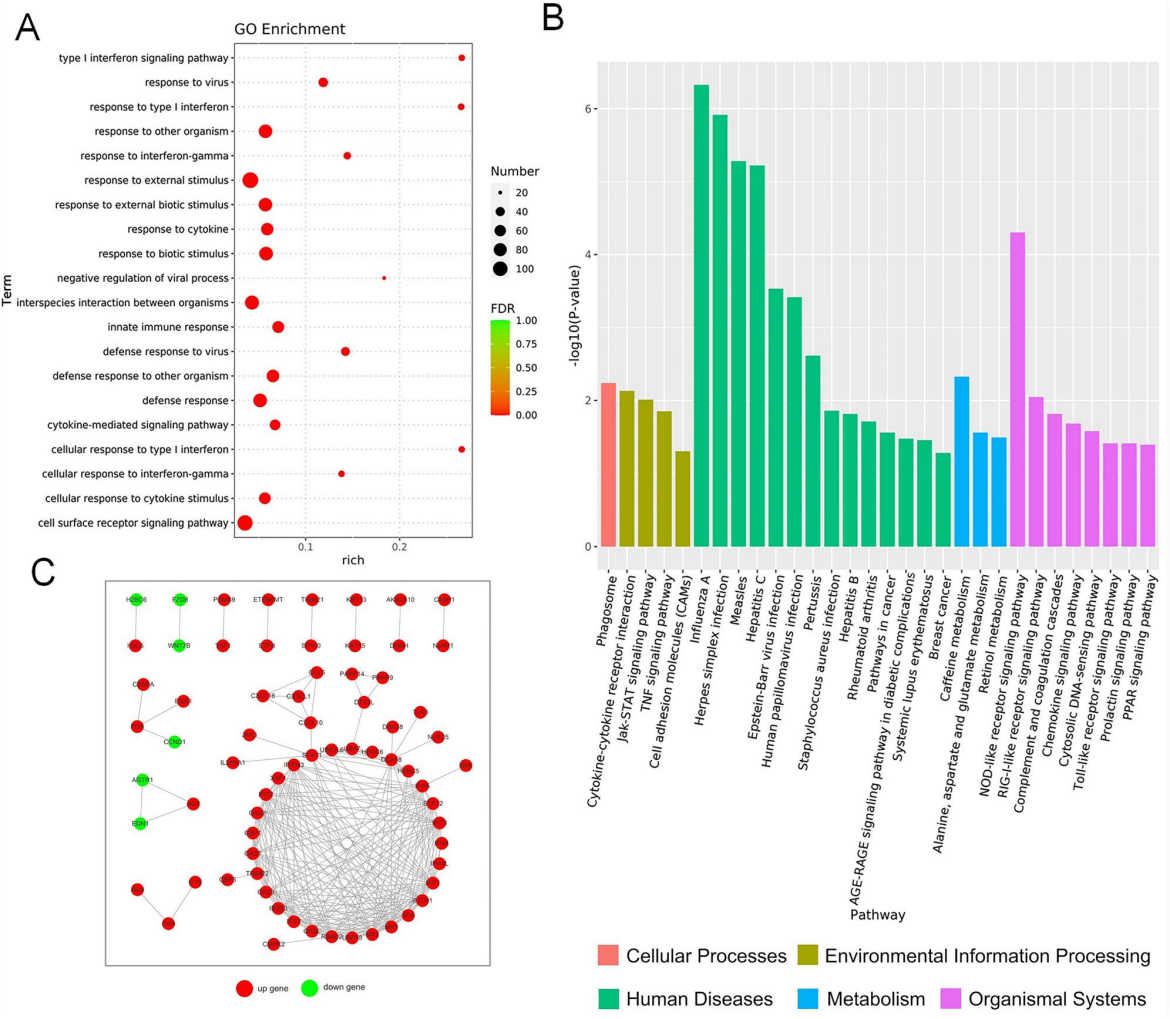
(**A**) GO enrichment of differentially expressed genes (DEGs) between temozolomide (TMZ) group and Control group. The abscissa is rich factor, which refers to the ratio of the number of differential genes enriched in the GO term to the number of genes annotated. The larger the rich factor, the greater the degree of enrichment. The ordinate is the enriched term. The color of bubbles represents the value of false discovery rate (FDR). The general value range of FDR is 0–1. The closer to zero, the more significant the enrichment is. The size of bubbles indicates the number of enriched differential genes. (**B**) Histogram of functional classified pathways between TMZ group and Control group. The abscissa is pathway name. The ordinate is P-value of each pathway. The different color of bars represents different functional classifications of pathways. (**C**) Network of DEGs involved between TMZ group and Control group. The dots are genes (corresponding proteins), where red represents up-regulated genes and green represents down regulated genes. The dots linked by gray lines are homologous proteins. The image (**A**) was generated by: topGO (v2.50.0), URL: https://www.bioconductor.org/packages/release/bioc/html/topGO.html. The image (**B**) was generated by: clusterProfiler (v4.6.0), URL: https://www.bioconductor.org/packages/release/bioc/html/clusterProfiler.html, images (**C**) was cited from STRING database, URL: https://string-db.org.

The expression level of 335 genes was changed compare to the control under 0.1 mg/ml TMZ treatment, in which 253 genes were increased and 82 genes were decreased (Fig. 2D). Viral infectious disease associated pathways and immune system pathway which overrepresented in Coix treatment group were also overrepresented in TMZ treatment group (Fig. 4A). The top eight enriched pathways with FDR less than 0.05 have 7 viral infectious disease associated pathways and one immune system pathway, and the top four of them were overlapping with the overrepresented pathways of Coix treatment group. The regulated genes in those pathways were also mostly overlapped with the Coix treatment group, however, with opposite regulation direction. IFN related genes such likeOAS2, RSAD2, OAS1, OAS3, DDX58, MX1, MX2, STAT1, CXCL10, TNFSF10 and IFIH1 which were down-regulated in Coix treatment group were up-regulated in TMZ treatment group compared to the control. It means that IFN related genes were induced by TMZ treatment and inhibited by Coix treatment. The induction of IFN related genes in TMZ treatment group was consistent with previous reports that TMZ treatment induces DNA mismatch and DNA damage caused by irradiation activates IFN. Therefore, the results indicating that TMZ treatment causes DNA damage in GBM cell and DNA damage activates the transcript of IFN related genes. Since IFN related genes were reported to have a certain impact on tumor proliferation, the inhibition of IFN related genes in Coix treatment group suggested that the induction of IFN related genes may relate to the mechanism of TMZ drug resistance, and the inhibition of these genes reverses drug resistance to TMZ.

### Dominate role of TMZ on interferon related genes’ regulation

**Figure 5:**
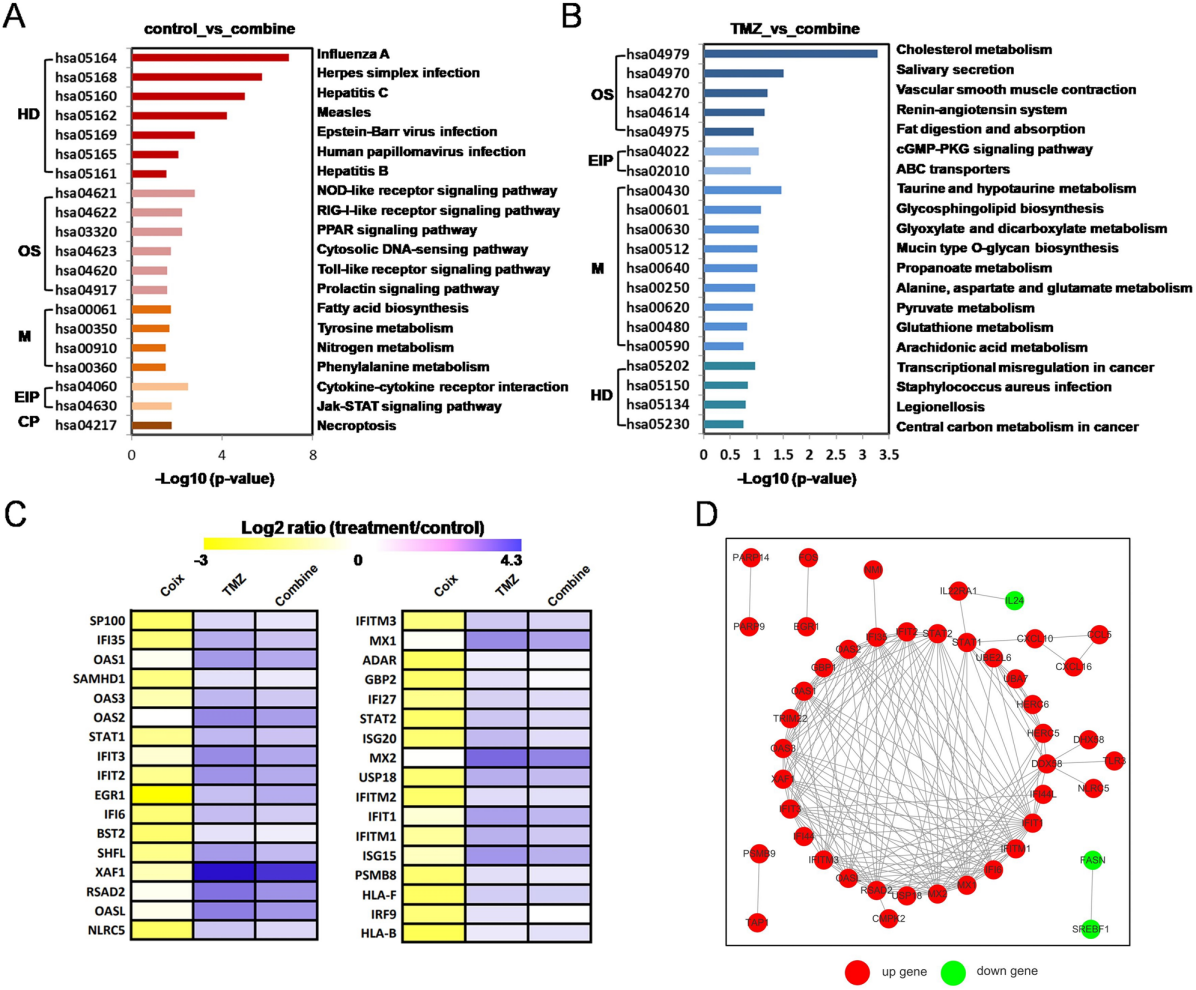
(**A**) Histogram of functional classified pathways between Combine group and Control group. The abscissa is p-value of each pathway. The ordinate is pathway name. The different color of bars represents different functional classifications of pathways. *HD* human diseases, *OS* organismal systems, *M* metabolism, *EIP* environmental information processing, *CP* cellular processes. (**B**) Histogram of functional classified pathways between Combine group and TMZ group. The abscissa is P-value of each pathway. The ordinate is pathway name. The different color of bars represents different functional classifications of pathways. *HD* human diseases, *OS* organismal systems, *M* metabolism, *EIP* environmental information processing, *CP* cellular processes. (**C**) Heatmap of differentially expressed IFN-I signaling genes. Each row represents a gene related to IFN-I, and each column represents an experimental group. The color of the square represents the ratio of gene expression to the control group. The more the color leans towards yellow, the lower the ratio. The more the color leans towards purple, the higher the ratio. (**D**) Network of differentially expressed genes (DEGs) involved between combine group and Control group. The dots are genes (corresponding proteins), where red represents up-regulated genes and green represents down regulated genes. The dots linked by gray lines are homologous proteins. The image (**A–C**) were generated by Excel (v2010), URL: http://best.zunsp.cn/?type=10&channel=dd-21, image (**D**) was cited from STRING database, URL: https://string-db.org.

Combination group have 295 DEGs compared to control group, among which 196 were up-regulated and 99 were down-regulated (Fig. 2D). Though inhibited by Coix treatment, IFN related genes in combined treatment group were still up-regulated as in TMZ treatment group, however, with a lower level. For example, RSAD2 which has a 2.37-fold down-regulation in Coix group was up-regulated 2.66- and 2.14-fold in TMZ group and combination group respectively. The four overrepresented KEGG enriched pathways (FDR < 0.05) in combination group were also identity to the top four in TMZ group (Fig. 5C). This result suggested a dominate role of TMZ in the combinatorial drug. Network of DEGs involved between TMZ group and control group is shown in Fig. 4C. Network of DEGs involved between combine group and control group is shown in Fig. 5D.

### Lipid related genes regulated by combinatorial drug

Despite the majority DEGs identities, TMZ group and combination group had 60 DEGs, many of which enriched in lipid related biological processes, such as GO: 0033993 response to lipid, GO: 0097006 regulation of plasma lipoprotein particle levels, GO: 0032526 response to retinoic acid, GO: 0071396 cellular response to lipid. This is consistent with the fact that Coix are an oil extract. The only stand out enriched KEGG pathway that had a FDR less than 0.05 between TMZ group and combination group was cholesterol metabolic pathway, which was also a lipid related pathway. In cholesterol metabolic pathway, ANGPTL4, which was able to activate ferroptosis that leading to cell apoptosis, was up-regulated in combination group compared to TMZ group, whereas ABCA1, which increases TMZ drug resistance by pumped TMZ out of the cell, was down-regulated. It indicated these genes played an important role in synergistic effect of the combinatorial drug.

### Verification of pathways

**Figure 6:**
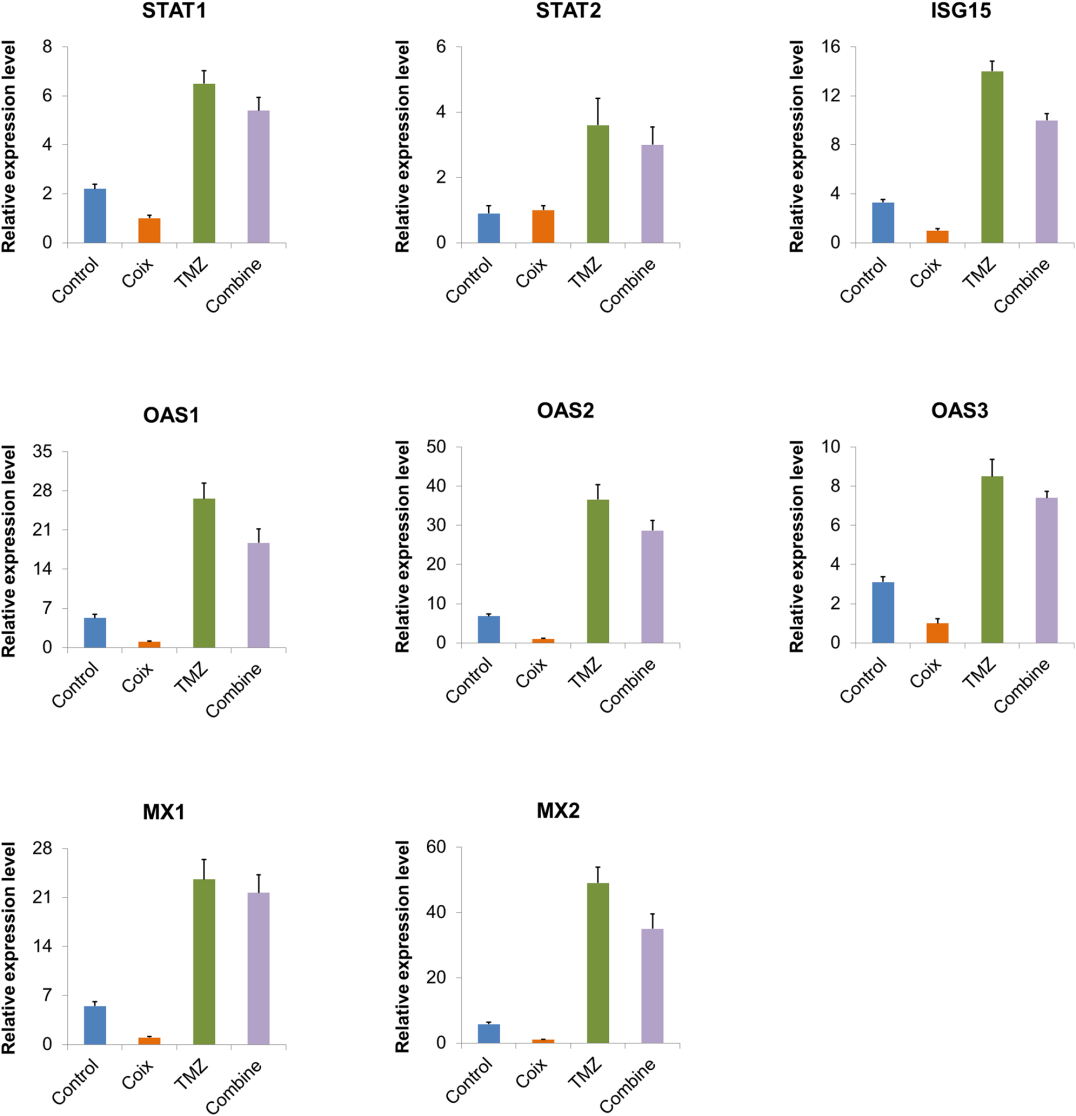
Histogram of key genes expression level in qRT-PCR. Each image shows the expression level of a key gene, and the name of the gene is labeled at the top of the image. The abscissa is group name. The ordinate is relative expression level. The images were generated by Excel (v2010), URL: http://best.zunsp.cn/?type=10&channel=dd-21.

The results of qRT-PCR shows that OAS1, OAS2, OAS3, MX1, MX2, STAT1, STAT2, ISG15 highly expressed in TMZ group. The above genes are low expressed in the COIX group. In the combine group, the gene expression was between the TMZ group and the Control group. This confirms that the IFN related pathway is activated in the TMZ group and inhibited in the Coix group. In the combine group, coix antagonized the activation of TMZ (Fig. 6).

## Discussion

Our results showed that Coix could inhibit the proliferation of GBM in vitro. The IC50 was 17.82 mg/ml and the maximum inhibition rate was 52.94%. TMZ has much stronger inhibitory ability on glioma, with IC50 of 0.358 mg/ml and the maximum inhibitory rate of 98.77%. Although Coix has weak inhibition ability, it can partially replace TMZ when combined with TMZ. Coix has been reported to be effective in improving patients’ symptoms and anticachexia^12^. Therefore, combined medication is of great significance to reduce the dosage of TMZ and reduce the side effects of chemotherapy. The combination of Coix and TMZ in small doses (TMZ 0.1 mg/ml, Coix 2 mg/ml) showed a synergistic effect on the inhibition of glioma growth. The transcriptome analysis applied on this dosage reveals interesting mechanisms on synergistic effect of TMZ and Coix.

IFN related genes were highlighted in transcriptome analysis. They were up-regulated by TMZ and down-regulated by Coix. The biology process that down-regulated DEGs enriched in between Coix group and control group are mainly IFN related, including processes directly related to IFN and processes indirectly related to IFN. Since IFN plays an important role in the process of virus resistance, virus infectious disease associated pathways and immune system pathways were overrepresented in the Coix group compared to the control. Most of these biology process and pathways were also overrepresented in TMZ group compared to the control, however, enriched with up-regulated DEGs.

Taking further investigation, we found the DEGs enriched in these pathways and biology process were mainly virus recognition and IFN response related. They sense and recognize abnormal genetic materials, such like viral dsRNA, and signaling to erect a line of defense. For example, IFIH1 which was up-regulated 2.7-fold by TMZ encodes a pattern recognition receptor that senses viral dsRNA and activates type I IFN antiviral signaling. IFN receptorJAK3 and signal transducers (STAT1 and STAT2) were also up-regulated. IFN induces an antiviral state of cells, and four groups of genes are induced to form this state of cells. All of them have members up-regulated by TMZ, including OAS1, OAS2 and OAS3 in OAS group, MX1 and MX2 in Mx group, ISG15 in ISG group. Besides IFN signal, the induction of the four group genes were also depend on abnormal genetic materials. PKRs and OASs are synthesized in an inactive form and utilize dsRNA as a cofactor. Mx proteins act by recognizing nucleocapsid-like structures^13^.

Why were IFN related pathways and biology process up-regulated by TMZ treatment? According to previous reports we described above, abnormal genetic materials are key affecters of IFN induction and response. The inductions of IFN related genes are depending on them. On the other hand, it is known that the mechanism of TMZ against glioma cells is DNA methylation, leading to gene mismatch accumulation and finally apoptosis^4^. TMZ induces DNA damage and the drug resistance was caused by the repairing of the damage. Thus, we suggest that the abnormal genetic materials caused by TMZ treatment induced DNA damage can be sensed by IFN related genes and activates antiviral IFN signaling, causing the up-regulation of IFN related genes and the overrepresented IFN related pathways and biology process in TMZ group compared to the control. This suggestion supported by studies on IFN induction that IFN related genes were up-regulated in cells after radiotherapy, and unrepaired DNA damage could induce the production of type I IFN^14^.

Most of IFN related genes that up-regulated by TMZ were down-regulated by Coix. We suppose the down-regulation of these genes act on reverse drug resistance of TMZ. The up-regulation of IFN related genes induced by TMZ treatment can reduce the effect of chemotherapy, which has been proved by relevant studies^15^. Under most situations, the STAT1/IFN pathway transmits a cytotoxic signal either in response to DNA damage or to IFNs. But under the chronic stimulation of DNA damage, it might have selected for the failure to transmit a cytotoxic signal and instead results in pro-survival signals mediated by STAT1 and other IFN-related DNA damage resistance signature genes^16, 17^. Previous studies also found that IFN not only play an important role in the process of virus resistance, but also have a certain impact on tumor proliferation. And IFN related genes are involved in tumor growth. For example, CXCL10 plays a key role in inhibiting glioma growth. Some studies have shown that the expression of CXCL10 is up-regulated in gliomas and increases with the increase of malignant degree of gliomas, and promotes early tumor growth by increasing tumor neovascularization^18–20^. IFIH1 can up-regulate the expression of CXCL10 by activating ISG56 through positive feedback^21^. RSAD2 and MAP2K6 play a key role in p38 MAP kinase signal cascade and may inhibit glioma proliferation by down-regulating p38 MAP pathway^22, 23^. In addition, IL-12 is up-regulated, which has been proved to have anti endogenous brain tumors in previous studies^24, 25^. Thus, the down-regulation of IFN related genes by Coix weakens the pro-survival signals making by IFN related genes, thereby, reverses the drug resistance of TMZ and causes synergistic effect of TMZ combined with Coix.

Besides IFN related genes, genes in cholesterol metabolism pathway were also highlighted in the study. Studies showed that ANGPTL4 promotes the migration, invasion and tubular formation of C6 cells in vitro. The tubular channel formed by it may play a role in the blood supply and metastasis of glioma. And ANGPTL4 can activate ferroptosis by activating NOX2^26, 27^. The fact that genes belong to ferroptosis were indeed regulated in the Coix group in our result supported the previous study. ABCA1 is a member of the ATP binding cassette (ABC) transporter superfamily^28^. Over-expression of ABCA1 can cause the mediated intracellular cholesterol imbalance, which may contribute to the growth and distant metastasis of primary tumors^29^. In the latest study, TMZ outflow was controlled by ABCA1 activity. The expression level of ABCA1 gene was an indicator of TMZ treatment efficiency and participated in the mechanism of TMZ resistance^30^. Therefore, the down-regulation of ABCA1 may be one of the reasons why Coix enhances the efficacy of TMZ.

## Conclusions

Coix had synergistic effect with TMZ when combined with TMZ at certain dose. The results of transcriptome analysis suggested that Coix inhibits drug resistance of TMZ by down-regulating IFN related genes. In addition, the cholesterol metabolic pathway may also enhance the effect of TMZ.

### Supplementary Information

Supplementary Information 1.

Supplementary Information 2.

Supplementary Information 3.

## Data Availability

The datasets generated during and/or analysed during the current study are available from the corresponding author on reasonable request. The raw data of transcriptome sequencing results in our experiment has been made available and uploaded to GSA database (https://ngdc.cncb.ac.cn/gsa-human/, Project ID: PRJCA012097). Data application address: https://bigd.big.ac.cn/gsa-human/browse/HRA003130 KEGG.

## Abbreviations

DMEM: Dulbecco’s Modified Eagle Medium
DMSO: Dimethylsulfoxide
D-PBS: Dulbecco’s phosphate-buffered saline
FBS: Fetal bovine serum
TMZ: Temozolomide
KLT: Kanglaite injection
CI: Combination index
DRI: Dose reduction index
CCK-8: Cell Counting Kit-8
EDTA: Ethylenediaminetetraacetic acid
