# Author Correction: Coixendide efficacy in combination with temozolomide in glioblastoma and transcriptome analysis of the mechanism

**DOI:** 10.1038/s41598-024-55383-0

**Published:** 2024-03-04

**Authors:** Zhenran Zhao, Lei Zhang, Xiaohan Zhang, Yong Yue, Shengchen Liu, Yanan Li, Xiang Ban, Cuizhu Zhao, Peng Jin

**Affiliations:** 1 Neurosurgery, The Affiliated Hospital of Qingdao University, Qingdao, 266000 Shandong China; 2 Neurosurgery, Linyi Traditional Chinese Medical Hospital, Linyi, 276000 Shandong China; 3 College of Agronomy, Northwest A&F University, Yangling, Xianyang, 712100 Shaanxi China

Correction to: *Scientific Reports* 10.1038/s41598-023-41421-w, published online 19 September 2023

The original version of this Article contained an error in the order of the author names, which was incorrectly given as Zhenran Zhao, Lei Zhang, Xiaohan Zhang, Yong Yue, Shengchen Liu, Yanan Li, Xiang Ban, Peng Jin & Cuizhu Zhao.

The original Article has been corrected.

