# Supplementary material for: Coixendide efficacy in combination with temozolomide in glioblastoma and transcriptome analysis of the mechanism
Source: Sci Rep. 2023 Sep 19;13:15484. doi: 10.1038/s41598-023-41421-w (PMC10509239; doi:10.1038/s41598-023-41421-w)
Supplement: Supplementary file 2 — Supplementary Information 2. [file 41598_2023_41421_MOESM2_ESM.pdf]

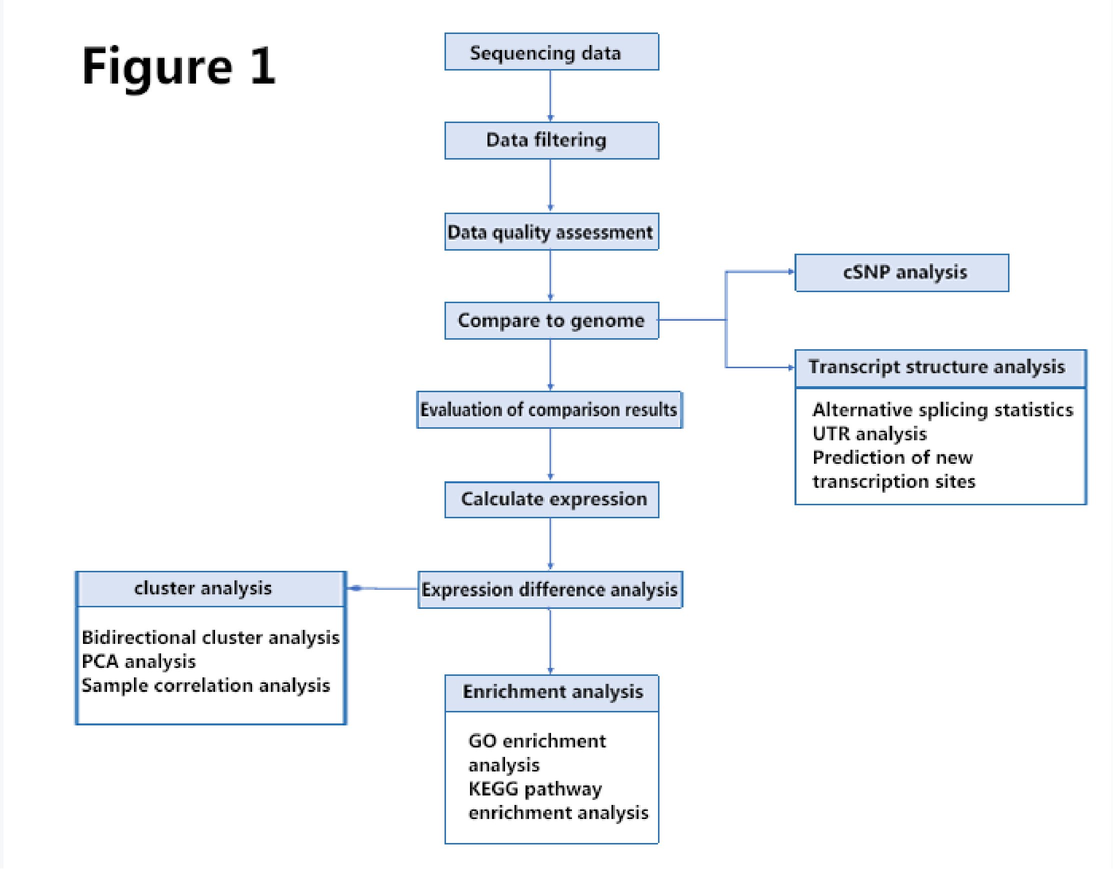

Supplementary figure 1
The workflow of RNA-seq and data analysis used in this study.

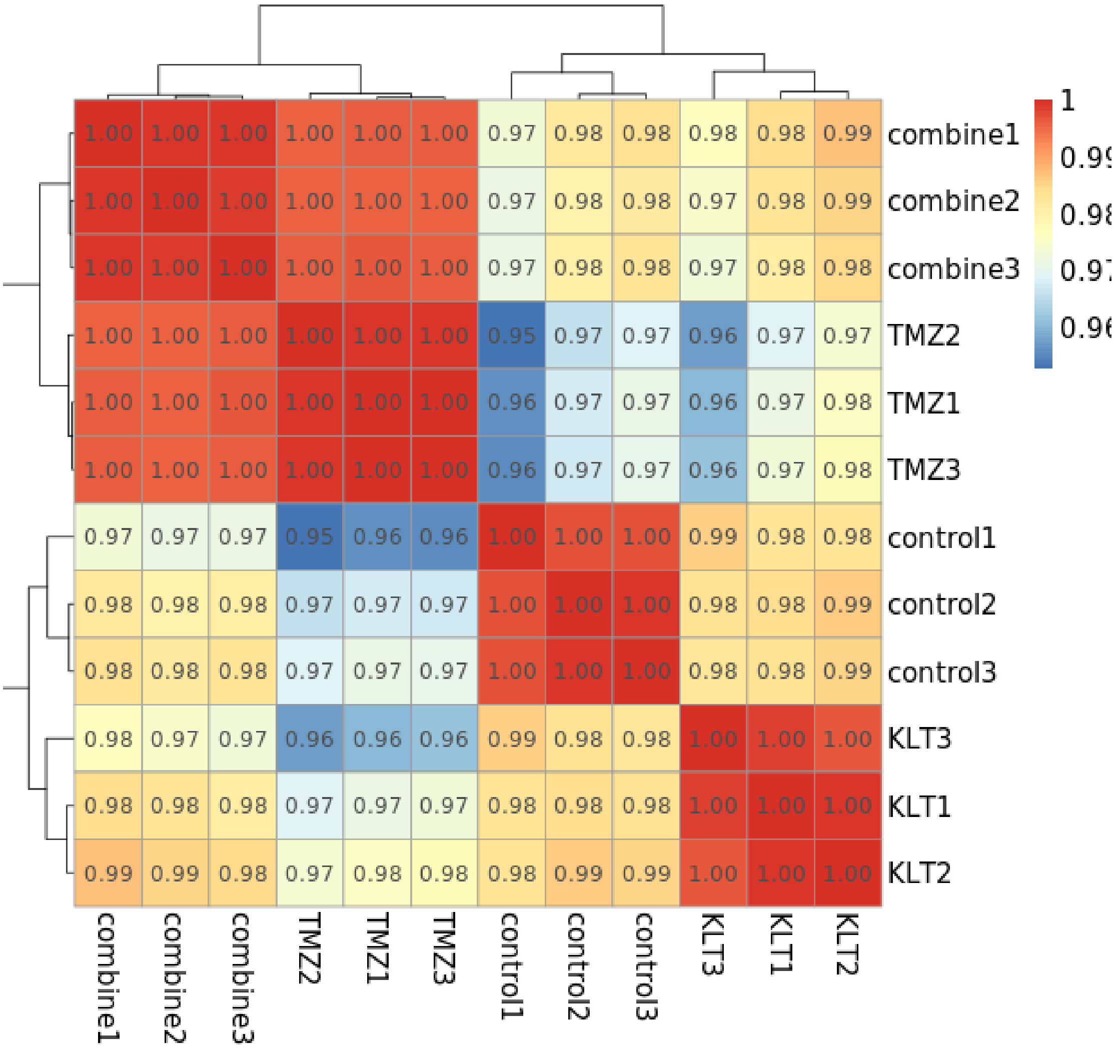

Supplementary figure 2

Correlation analysis of samples.

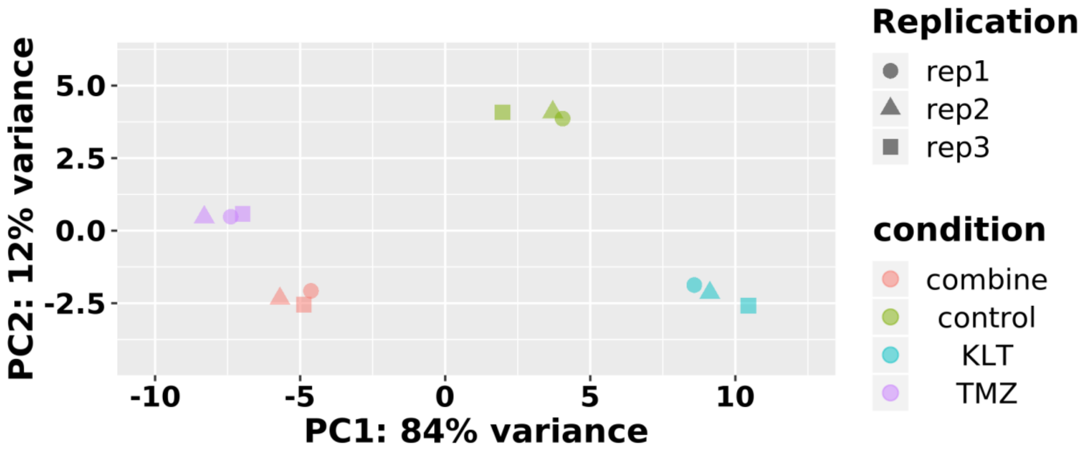

Supplementary figure 3

PCA analysis of samples.

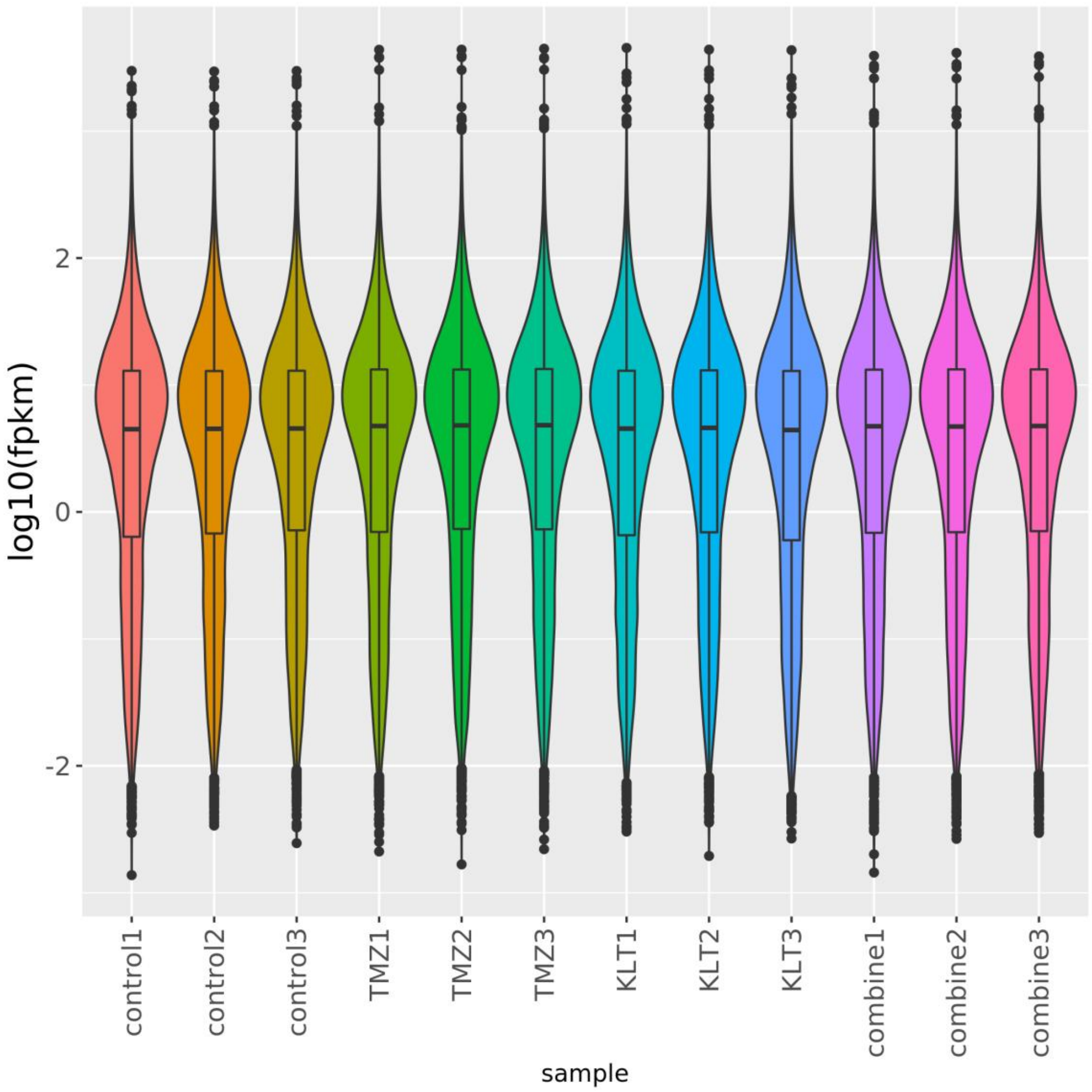

Supplementary figure 4
Violin plot of samples.

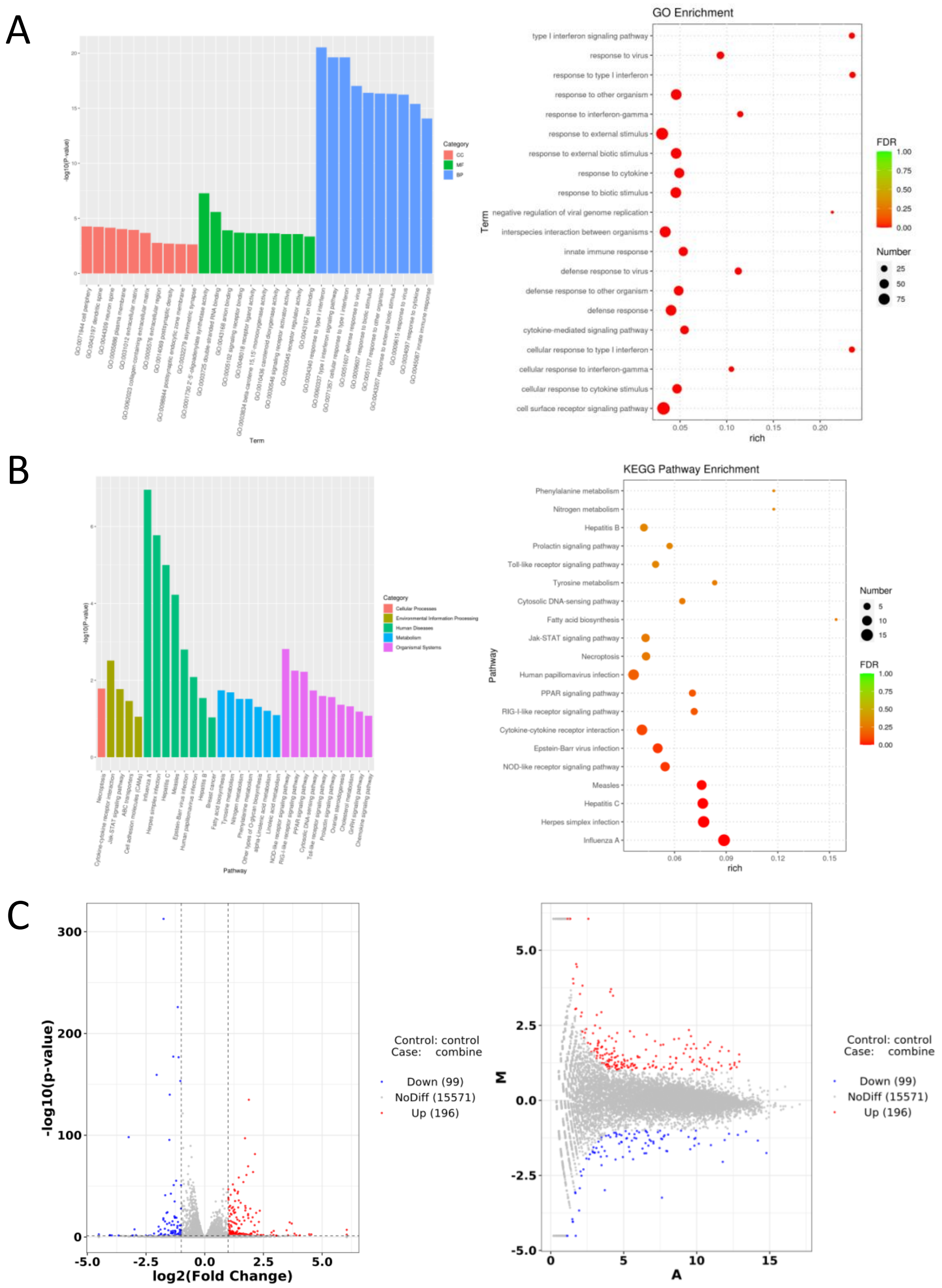

Supplementary figure 5

Analysis of differentially expressed genes (DEGs) between combine group and control group. (A) GO enrichment of DEGs between combine group and control group. Top 20 enriched GO terms were shown here. (B) KEGG pathway enrichment of DEGs between combine group and control group. Top 20 enriched pathways were shown here. (C) Volcano and MA blot showing differentially expressed genes (DEGs) between combine group and control group.

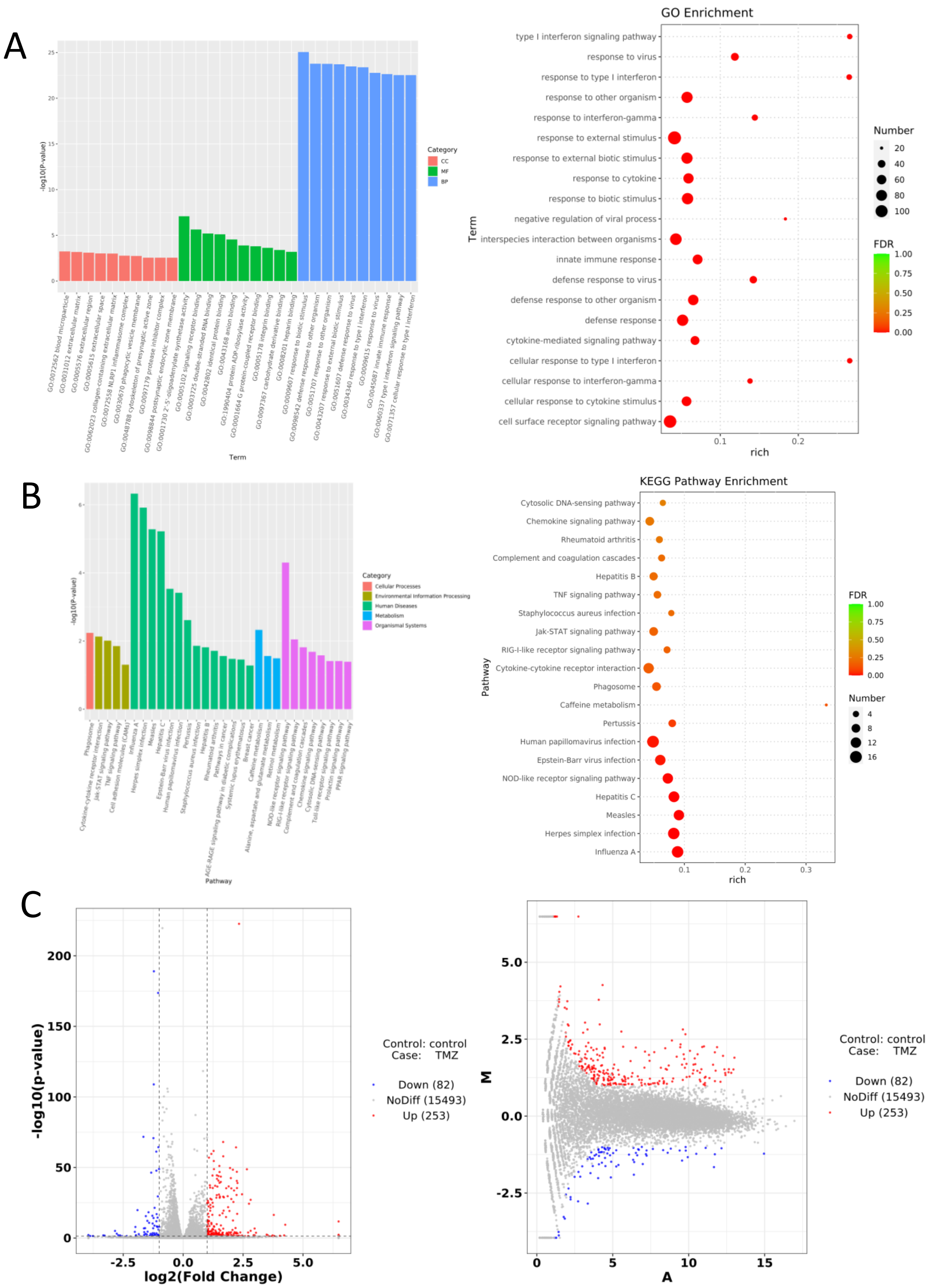

Supplementary figure 6

Analysis of differentially expressed genes (DEGs) between TMZ group and control group. (A) GO enrichment of DEGs between TMZ group and control group. Top 20 enriched GO terms were shown here. (B) KEGG pathway enrichment of DEGs between TMZ group and control group. Top 20 enriched pathways were shown here. (C) Volcano and MA blot showing differentially expressed genes (DEGs) between TMZ group and control group.

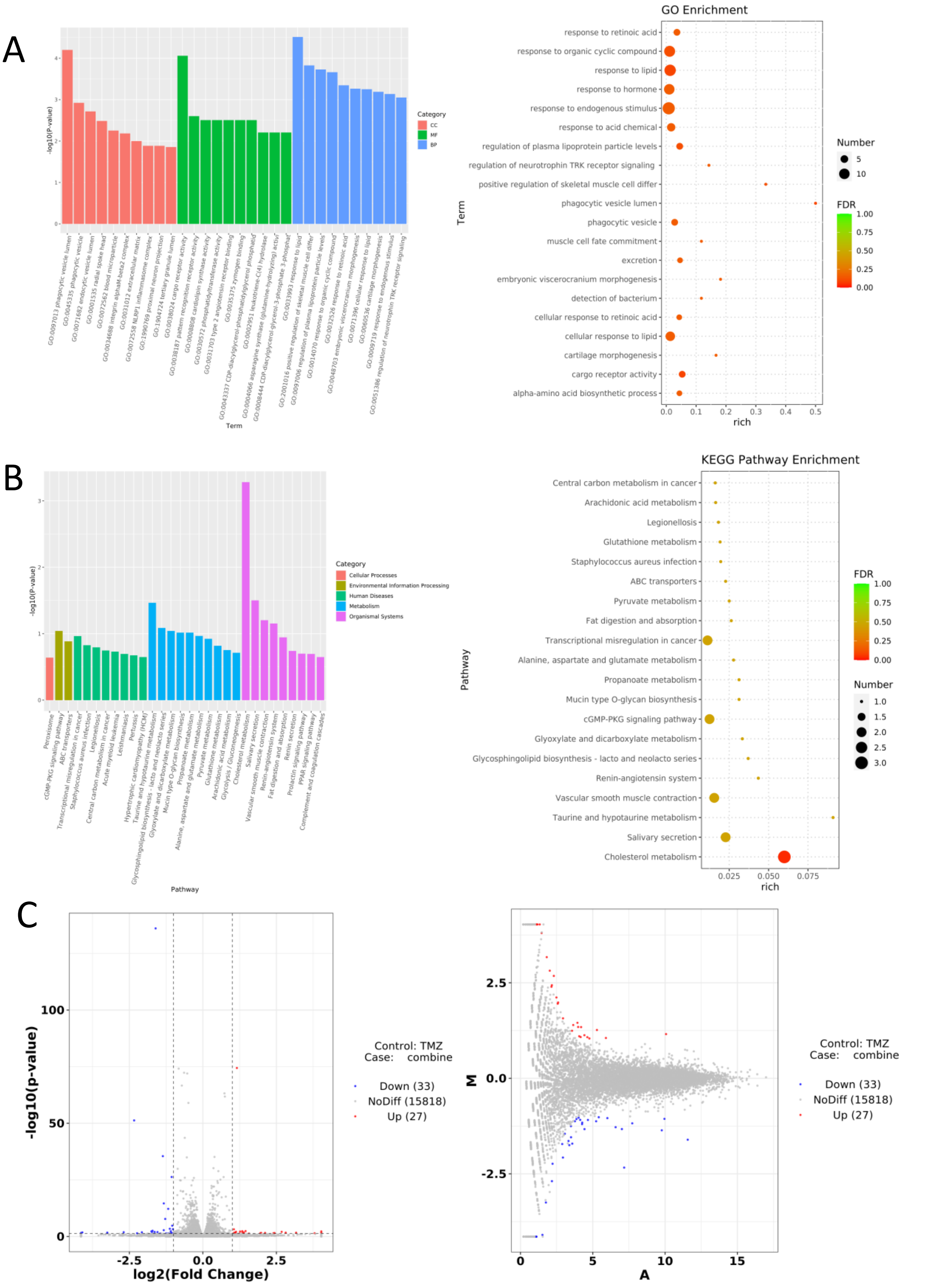

Supplementary figure 7

Analysis of differentially expressed genes (DEGs) between TMZ group and combine group. (A) GO enrichment of DEGs between TMZ group and combine group. Top 20 enriched GO terms were shown here. (B) KEGG pathway enrichment of DEGs between TMZ group and combine group. Top 20 enriched pathways were shown here. (C) Volcano and MA blot showing differentially expressed genes (DEGs) between TMZ group and combine group.

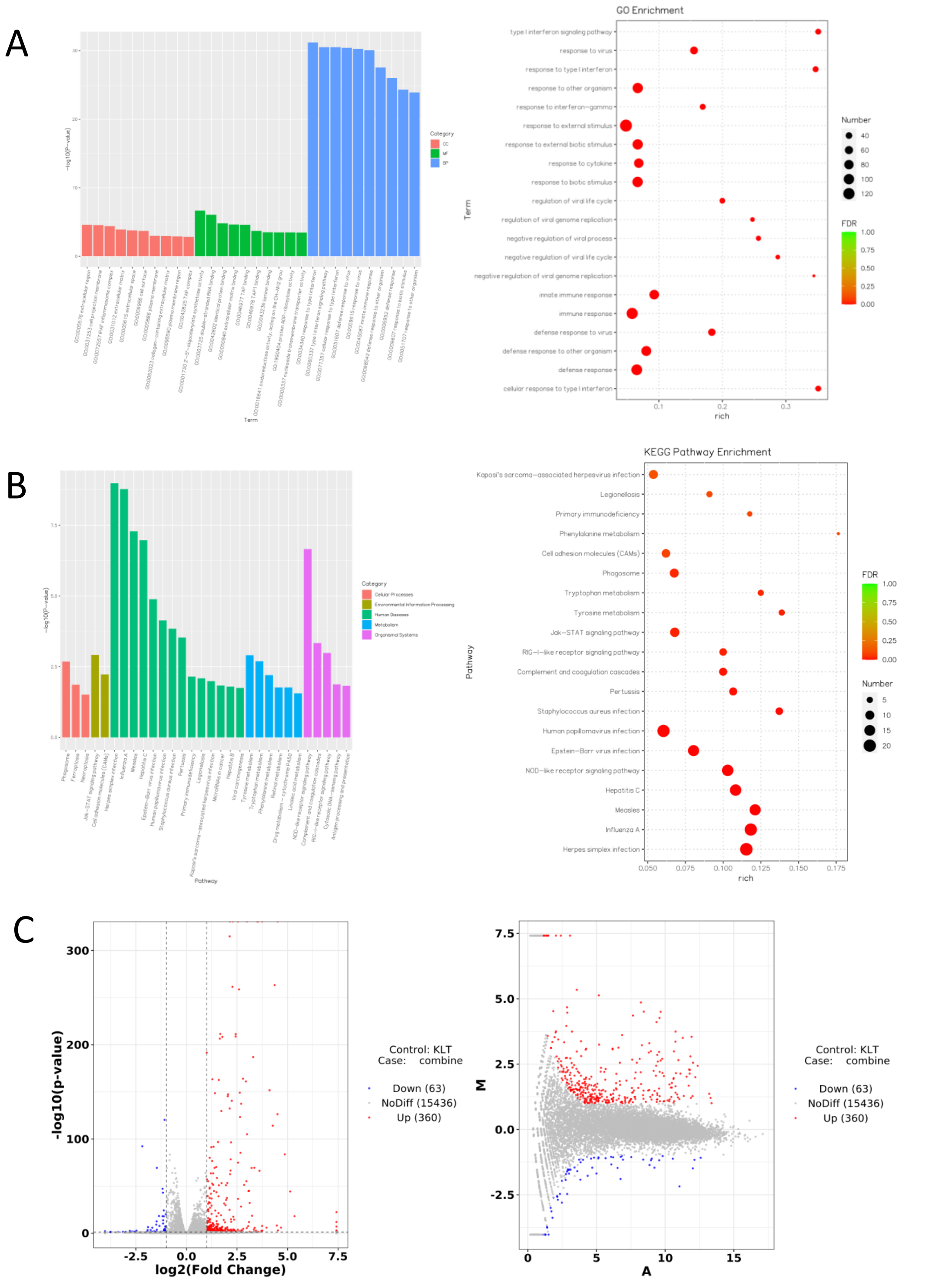

Supplementary figure 8

Analysis of differentially expressed genes (DEGs) between Coix group and combine group. (A) GO enrichment of DEGs between Coix group and combine group. Top 20 enriched GO terms were shown here. (B) KEGG pathway enrichment of DEGs between Coix group and combine group. Top 20 enriched pathways were shown here. (C) Volcano and MA blot showing differentially expressed genes (DEGs) between Coix group and combine group.
